# Differences in nutrient composition and choice of side dishes between red meat and fish dinners in Norwegian adults

**DOI:** 10.3402/fnr.v60.29555

**Published:** 2016-01-14

**Authors:** Jannicke Borch Myhre, Elin Bjørge Løken, Margareta Wandel, Lene Frost Andersen

**Affiliations:** Department of Nutrition, University of Oslo, Oslo, Norway

**Keywords:** red meat, fish, dinner, side dish, macronutrients

## Abstract

**Background:**

Food-based dietary guidelines often recommend increased consumption of fish and reduced intake of red and processed meat. However, little is known about how changing the main protein source from red meat to fish may influence the choice of side dishes.

**Objective:**

To investigate whether side dish choices differed between red meat and fish dinners. Moreover, to compare intakes of macronutrients and selected micronutrients in red meat and fish dinners and to see whether whole-day intakes of these nutrients differed between days with red meat dinners and days with fish dinners.

**Design:**

Data were collected in a cross-sectional nationwide Norwegian dietary survey using two non-consecutive telephone-administered 24-h recalls. The recalls were conducted approximately 4 weeks apart. In total, 2,277 dinners from 1,517 participants aged 18–70 were included in the analyses.

**Results:**

Fish dinners were more likely to include potatoes and carrots than red meat dinners, whereas red meat dinners more often contained bread, tomato sauce, and cheese. Red meat dinners contained more energy and iron; had higher percentages of energy (E%) from fat, saturated fat, and monounsaturated fat; and a lower E% from protein and polyunsaturated fat than fish dinners. Fish dinners contained more vitamin D, β-carotene, and folate than red meat dinners. Similar differences were found when comparing whole-day intakes of the same nutrients on days with red meat versus fish dinners.

**Conclusion:**

Fish dinners were accompanied by different side dishes than red meat dinners. With regard to nutrient content, fish dinners generally had a healthier profile than red meat dinners. However, iron intake was higher for red meat dinners. Information about associated foods will be useful both for developing public health guidelines and when studying associations between dietary factors and health outcomes.

The consumption of red or processed meat has been associated with various adverse health outcomes, such as diabetes type 2 (1), cardiovascular disease (1), and cancer, particularly colorectal cancer (2). A higher intake of red meat, in particular processed red meat, has also been associated with higher all-cause mortality (3). Several food-based dietary guidelines (FBDGs) emphasize the need for reducing the intake of red/processed meat (4). Fish represents an alternative protein source to meat, and increased consumption of fish is often recommended by FBDGs (4). Also the report ‘Food, Nutrition, Physical Activity, and the Prevention of Cancer: a Global Perspective’ from 2007 recommends replacing red meat with fish or poultry (2). In Norway, fish consumption is higher than in several other Western countries (5). Nevertheless, meat consumption is still substantially higher than fish consumption, both according to previous Norwegian dietary surveys (6, 7) and household budget surveys. Although the Norwegian household budget survey from 2012 showed that 35 g of fish (including shellfish) was purchased per person per day, the corresponding figure for meat was 131 g per person per day (8). The Norwegian FBDGs recommend an intake of 300–450 g fish per week of which at least 200 g should be oily fish (9). The most recent Norwegian dietary survey in adults, Norkost 3, showed that only 18% of the participants had an intake matching this recommendation (10). Hence, a large proportion of the population would benefit from an increased fish intake. However, because a dinner meal most often contains more than just a protein component, side dish choice is also important to the complete nutritional profile of the meal in question. Changing the main protein source of a meal (e.g. from meat to fish) may also lead to alterations in side dish choice. These changes may increase or decrease the nutritional gain in quality, depending on the compositions of the chosen side dishes. The aim of the present study was to investigate whether side dish choice varied for red meat versus fish dinners in a group of Norwegian adults. We also compared the intakes of macronutrients and selected micronutrients in red meat and fish dinners and assessed whether the whole-day intakes of these nutrients differed on days when each type of dinner was consumed. Older individuals in the Norwegian population consume more fish than younger age groups (10, 11), and side dish choices may also vary according to age. Therefore, the analyses of differences in side dish choices between red meat and fish dinners were stratified according to age.

## Methods

### Subjects and design

**Fig. 1:**
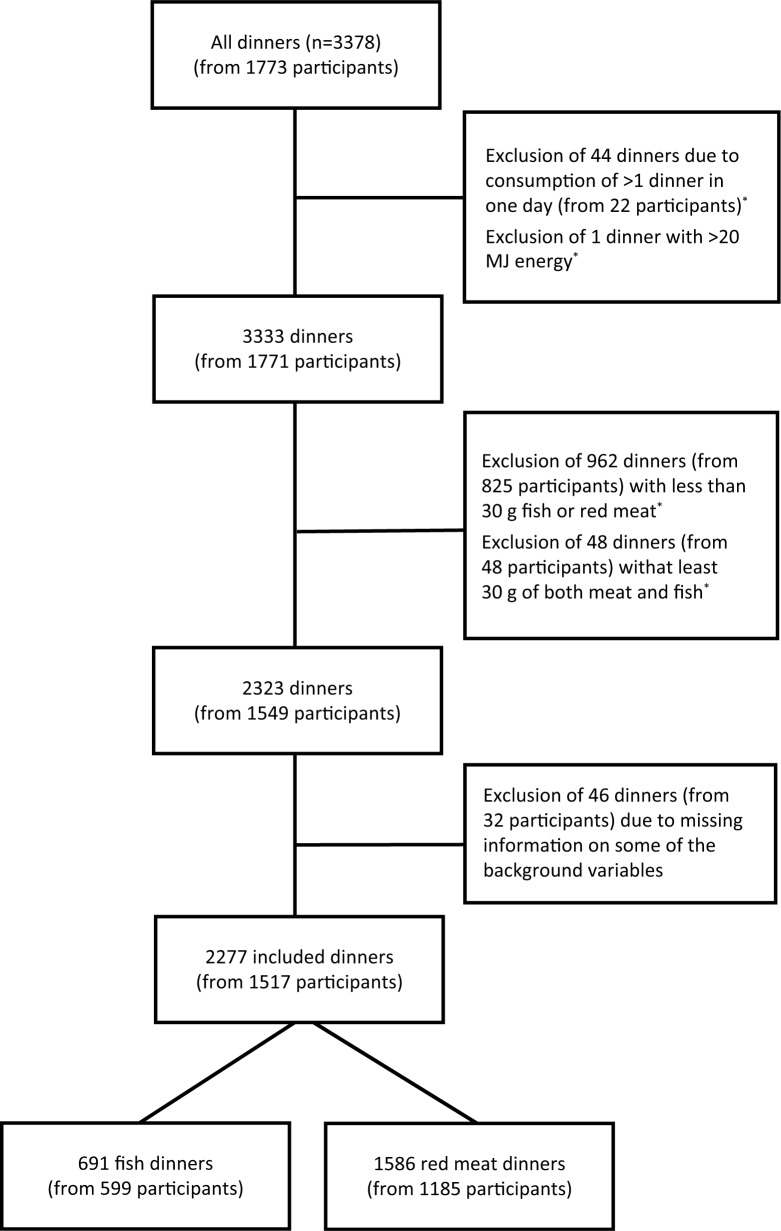
Flow chart of inclusion and exclusion of dinners recorded in the Norkost 3 study, 2010–2011. *The participant could still participate with the dinner from the other recall day if it was eligible for inclusion.

The data for the present study were obtained from Norkost 3, a Norwegian dietary survey conducted in 2010–2011. The design and methodology have been described in detail elsewhere (12). A representative sample (*n*=5,000) of the Norwegian population aged 18–70 years was randomly selected from the National Register and asked to complete two telephone-administered 24-h recalls approximately 4 weeks apart. Because of experiences of language barriers and very low participation rates among non-Western immigrants from a pilot study conducted prior to the Norkost 3 study, only persons born in Norway, Sweden, and Denmark were contacted. Of them, 153 were unsuitable for participation (wrong phone number, not Norwegian, or accidentally invited to participate twice). Of the remaining 4,847 suitable invitees, 1,787 participants completed two 24-h recalls, resulting in a participation rate of 37%. Figure 1 shows the details of the inclusion and exclusion of dinners. In total, 2,277 eligible dinners from 1,517 participants were included in the analyses. This study was conducted according to the guidelines established in the Declaration of Helsinki. All procedures involving human subjects were approved by the Regional Committee for Medical and Health Research Ethics. Verbal informed consent was obtained from all participants.

### Assessment of dietary intake

The 24-h recalls aimed to include all foods and beverages consumed in the period between waking on the preceding day and waking on the interview day. Interviews were conducted by trained personnel using an in-house data program (KBS v7.0) linked directly to a food composition database. This food composition database was based on the Norwegian Food Composition Table from 2006 (13) and supplemented with additional food items from reliable sources. Before starting the recall, the participant was asked whether the previous day was normal with regard to food and beverage intakes (yes/no). Seventy-three percent of the days were considered normal. During the recall, each eating or drinking occasion was defined by the respondent as breakfast, lunch, dinner, supper, or snack. For the present analyses, only eating occasions defined as ‘dinner’ were included. All components of the dinner meals were included, such as beverages, appetizers, and desserts.

### Food group definitions

Red meat was defined as muscle and organ meat from pork, beef, mutton, and goat (9), whereas fish was defined as all fish and fish products, excluding shellfish. Food weights are provided as grams of prepared meat or fish. Meat products, such as meatballs and sausages, and fish products, such as fish cakes, were regarded as 100% red meat or fish, respectively, even though other ingredients may have been added. This approach was chosen because such products are normally regarded as one unit.

For composite dishes, such as pizza or fish au gratin, the dish was broken down into its main constituents. For example, pizza was broken down into pizza crust (grouped as bread), meat, tomato sauce, vegetables, and cheese, whereas fish au gratin was considered as fish, pasta, and white sauce.

For the comparison of side dish use between the red meat and fish dinners, side dishes considered to be common components of dinners were included in the analyses. Foods or food groups consumed by a low proportion of participants (<10% in all age groups) were not included in this comparison. The food group ‘bread’ included regular bread, hamburger buns, hot dog buns, pizza crusts, crisp bread, tortillas, taco shells, and salty crackers. ‘Rice’ included all types of boiled rice, whereas ‘pasta’ included boiled pasta and noodles. ‘Boiled potatoes’ included all types of boiled potatoes. Fried potatoes, French fries, and other potato dishes, such as mashed and gratinated potatoes, are not presented because a low percentage of participants had consumed these dishes. ‘Vegetables’ included fresh, frozen, and canned vegetables (excluding dry legumes), but not those vegetables that are included in tomato sauces as tomato sauce is presented as a separate food group including tomato sauces used on pizza, pasta, tacos, and casseroles, as well as ketchup and salsa. Sauces other than tomato sauce were grouped according to fat content. Finally, the food group ‘cheese’ included cheeses used for cooking, such as those on pizzas, burgers, and pasta.

### Inclusion of eligible dinners

Red meat dinners were defined as dinners containing at least 30 g of prepared red meat and <30 g of prepared fish or fish products. Fish dinners were defined as dinners containing at least 30 g of prepared fish or fish products and <30 g of prepared meat or meat products. We chose to include all types of meat and not only red meat in the 30-g upper limit for fish dinners to include dinners in which fish was the main protein component rather than meat products not defined as red meat, such as poultry or game. The 30-g limit was set to include dinners containing at least a meat or fish portion corresponding to approximately half of a sausage or half of a fish cake. Dinners containing <30 g of either fish or red meat were not included in the analyses.

### Background variables

The participants were categorized into three age groups: 18–34, 35–54, and 55–70 years. Body mass index (BMI) was calculated based on self-reported weight and height as weight (kg) divided by height squared (m^2^) and dichotomized into ‘normal weight (BMI <25.0 kg/m^2^)’ and ‘overweight (BMI ≥25.0 kg/m^2^)’. Education level was originally grouped into eight categories, ranging from ‘no education’ to ‘university/college education at masters/PhD level’, but was regrouped to ‘high school, technical school, trade school, or lower’ and ‘university or college education’. Smoking habits were originally grouped into three categories but were regrouped to ‘smokers (daily/occasional smokers)’ and ‘non-smokers (never-smokers or previous smokers)’. Interest in a healthy diet was originally grouped into five categories, ranging from ‘no interest’ to ‘very high interest’, but was regrouped to ‘no, low, or moderate interest’ and ‘high or very high interest’. Weekdays were defined as Monday through Thursday and weekends as Friday through Sunday.

### Statistical analyses

Statistical analyses were carried out using Stata v13.1 (StataCorp, College station, Texas). As results did not differ between men and women, the analyses were not stratified by sex. All tests were two-sided. As multiple observations were available for many participants because of consumption of more than one dinner (i.e. a red meat or a fish dinner was consumed on both recall days), mixed models with a random effect (random intercept) for participant were used to adjust for this dependency in the data. Because of the large number of non-consumers for each of the presented side dishes, the continuous side dish variables were dichotomized into dinners containing/not containing each of the side dishes, and the results are presented as percentages of dinners containing each side dish (Table 2). For the comparison of the percentages of red meat and fish dinners containing each side dish, a logistic mixed model was used with using or not using each of the side dishes as dependent variables. These models were adjusted for sex, BMI, educational level, interest in a healthy diet, smoking habits, whether the day was a normal day with regard to food and beverage intakes, and weekday versus weekend. Because of the multiple comparisons, the significance level was set to *p*<0.003 (*p*<0.05 divided by 18 tests, one for each side dish per age group).

For the comparisons of nutrient intakes between the red meat and fish dinners (Table 3), mixed models with energy intake and each of the listed nutrients as dependent variables were used. The intakes of protein, fat, fatty acids, carbohydrates, and added sugars are presented as the percentage of energy intake from each nutrient. Fiber intake is presented as grams per megajoule (MJ). Because of many non-consumers, alcohol intake was dichotomized into dinners containing/not containing alcohol, and the results are presented as percentages of dinners containing alcohol. The intakes of iron, folate, vitamin C, β-carotene, and vitamin D are presented as absolute intakes per dinner. Linear mixed models were used for all analyses except for alcohol, for which a logistic mixed model was used. The models were adjusted for sex, age group, BMI, educational level, interest in a healthy diet, smoking habits, whether the day was a normal day with regard to food and beverage intakes, and weekday versus weekend. Because of the multiple comparisons, the significance level was set to *p<*0.003 (*p<*0.05 divided by 15 tests).

Mixed models were also used for the comparison of the whole-day intakes of the same nutrients on the days with red meat versus fish dinners (Table 4). Alcohol intake was dichotomized into consumer/non-consumer as described above. These models were adjusted for the same independent variables as those comparing the individual dinners, and the significance level was set to *p<*0.003 as for the previous models.

## Results

In total 691 fish dinners and 1,586 red meat dinners of 1,517 participants were included (Fig. 1). Table 1 shows selected background characteristics of the participants contributing either red meat dinners, fish dinners, or one of each to the analyses.

**Table 1:** Background characteristics of participants consuming dinner(s) with a minimum of either 30 g of red meat or 30 g of fish, Norkost 3 study, 2010–2011

	Participants (*n*=1,517)	
	
	*N*	%
Sex		
Male	763	50
Female	754	50
Age group		
18–34 years	330	22
35–54 years	695	46
55–70 years	492	32
BMI		
BMI<25.0 kg/m^2^	754	50
BMI≥25.0 kg/m^2^	763	50
Educational level		
High school or lower	719	47
University/college	798	53

BMI, body mass index.

Table 2 shows the percentages of red meat and fish dinners containing various side dishes in three age groups. Across age groups, bread was more commonly consumed with red meat than fish dinners, whereas the opposite was observed for boiled potatoes. Vegetables were more commonly eaten with fish than red meat dinners, although the difference was only statistically significant in the middle-aged group (35–54 years, *p<*0.001). We also looked at the total amounts of vegetables consumed for each dinner type. A statistically significant difference between red meat and fish dinners was seen only in the youngest age group in which red meat dinners contained 73 g of vegetables and fish dinners contained 107 g of vegetables (*p*=0.001). For the middle-aged group, red meat dinners were accompanied by 96 g of vegetables, whereas fish dinners contained 107 g vegetables (*p*=0.09). Finally, in the oldest age group, red meat dinners contained 106 g vegetables, whereas fish dinners contained 119 g vegetables (*p*=0.08). It should be noted, however, that vegetables included in tomato sauces were not included in the sum of vegetables. When combining the amount of tomato sauce and vegetables, no differences were seen in total amounts of vegetables and tomato sauce between red meat and fish dinners for either age group. Some distinct patterns were found regarding choice of vegetables; carrots were included in at least 50% of fish dinners for all age groups, whereas only 15–27% of red meat dinners contained carrots. However, lettuce was more commonly consumed with red meat than fish. This difference was statistically significant in the middle-aged group (*p*<0.001) and approached significance in the oldest age group (*p*=0.007). With regard to sauce, the largest differences between the fish and red meat dinners were observed for tomato sauce; in the youngest age group, 44% of red meat dinners and only 5% of fish dinners contained tomato sauce (*p*<0.001). Cheese was also more often consumed with red meat dinners, particularly in the youngest age group.

**Table 2:** Percentages of red meat and fish dinners containing various side dishes with results stratified according to age group (*n*=1,517 participants)

	Age 18–34 years (*n*=330)			Age 35–54 years (*n*=695)			Age 55–70 years (*n*=492)		
			
	Red meat dinner^a^ (382 dinners)	Fish dinner^a^ (101 dinners)		Red meat dinner^a^ (731 dinners)	Fish dinner^a^ (292 dinners)		Red meat dinner^a^ (473 dinners)	Fish dinner^a^ (298 dinners)	
				
Food/food group	% of dinners	% of dinners	*p* b	% of dinners	% of dinners	*p* b	% of dinners	% of dinners	*p* b
Bread^c^	41	11	<0.001	34	12	<0.001	22	11	0.003
Rice	9	20	0.009	8	12	0.033	5	4	0.55
Pasta	20	8	0.006	14	10	0.014	10	10	0.89
Potato, boiled	18	48	<0.001	29	56	<0.001	51	70	<0.001
Vegetables, all	62	77	0.014	70	86	<0.001	76	84	0.042
Carrot	15	50	<0.001	22	52	<0.001	27	61	<0.001
Broccoli	8	18	0.007	11	19	0.005	12	18	0.09
Onion	26	21	0.15	22	20	0.68	20	19	0.87
Corn	13	10	0.86	12	8	0.07	7	6	0.54
Bell pepper	18	12	0.10	18	7	<0.001	11	8	0.20
Lettuce	21	10	0.017	23	10	<0.001	16	9	0.007
Cucumber	11	13	0.48	15	12	0.38	12	9	0.24
Tomato	17	14	0.56	21	11	<0.001	18	11	0.026
Sauce<5% fat	16	17	0.90	18	12	0.017	24	9	<0.001
Sauce 5–15% fat	16	17	0.76	19	19	0.97	18	19	0.59
Sauce>15% fat	4	12	<0.001	5	7	0.07	4	6	0.10
Tomato sauce	44	5	<0.001	32	3	<0.001	18	1	<0.001
Cheese	34	9	<0.001	22	7	<0.001	12	3	0.001

a Red meat dinners were defined as dinners containing at least 30 g of red meat and less than 30 g of fish, while fish dinners were defined as dinners containing at least 30 g of fish and less than 30 g of meat.

b Logistic mixed model with consuming/not consuming the food group in question as the dependent variable, adjusted for sex, BMI, educational level, interest in a healthy diet, smoking habits, whether the day was normal with regard to food and beverage intake, and weekdays/weekends.

c Includes regular bread, hamburger buns, hot dog buns, pizza crusts, crisp bread, tortillas, taco shells, and salty crackers. The significance level was set to *p<*0.003 due to multiple testing.

Bread (i.e. pizza crust), tomato sauce, and cheese are typical components of pizzas. Therefore, we performed a subanalysis of red meat dinners containing bread to see how many of them consisted of pizza. In total 510 red meat dinners contained bread, and of these 19% consisted of pizza. The percentage of all red meat dinners being pizza was 8, 6, and 4% in the youngest, middle-aged, and oldest age groups, respectively. None of the fish dinners consisted of pizza.

The comparison of energy and nutrient intakes between fish and red meat dinners showed that fish dinners contained less energy and had a higher percentage of energy from protein than meat dinners (Table 3). Dietary fiber in grams/megajoule was also somewhat higher in fish dinners. Fish dinners had lower percentages of energy from total fat, saturated fatty acids (SFAs), and monounsaturated fatty acids (MUFAs) and a higher percentage of energy from polyunsaturated fatty acids (PUFAs) than red meat dinners. The intakes of β-carotene, folate, and vitamin D intake were higher for fish than red meat dinners, whereas iron intake was higher for red meat dinners. Differences in whole-day energy and nutrient intakes on days with fish dinners versus days with red meat dinners were similar to those observed for the comparison of the dinners alone (Table 4).

**Table 3:** Macronutrient composition and content of selected micronutrients in red meat and fish dinners (*n*=1,517 participants)

	Red meat dinner^a^ (1,586 dinners)		Fish dinner^a^ (691 dinners)		
			
Intake from dinner	Mean^c^	95% CI	Mean^c^	95% CI	*p* b
Energy, MJ/dinner	3.4	3.4,3.5	2.9	2.8,3.1	<0.001
Protein, E%	21	21,22	27	27,28	<0.001
Fat, E%	40	40,41	35	34,36	<0.001
SFAs, E%	16	16,16	11	11,12	<0.001
MUFAs, E%	15	15,16	11	11,12	<0.001
PUFAs, E%	6	6,6	8	8,8	<0.001
Carbohydrates, E%	35	34,35	34	33,35	0.13
Added sugar, E%	5	4,5	4	3,4	<0.001
Fiber, g/MJ	2.1	2.1,2.2	2.4	2.3,2.5	<0.001
Alcohol, % dinners containing	12	–	10	–	0.40
Vitamin D, µg/dinner	1	0,1	8	7,8	<0.001
Folate, µg/dinner	76	73,78	92	87,96	<0.001
Vitamin C, mg/dinner	43	41,45	42	38,45	0.53
β-carotene, µg/dinner	1,414	1,281,1,548	3,168	2,967,3,367	<0.001
Iron, mg/dinner	5	5,5	3	2,3	<0.001

MJ, mega joule; E%, percentage of energy; SFAs, saturated fatty acids; MUFAs, monounsaturated fatty acids; PUFAs, polyunsaturated fatty acids.

a Red meat dinners were defined as dinners containing at least 30 g of red meat and less than 30 g of fish, while fish dinners were defined as dinners containing at least 30 g of fish and less than 30 g of meat.

b Linear mixed models for continuous variables and logistic mixed model for alcohol use.

c Means and 95% CIs adjusted for sex, age group, BMI, educational level, interest in a healthy diet, smoking habits, whether the day was normal with regard to food and beverage intake, and weekdays/weekends. For alcohol, the percentage of dinners with alcohol intake is presented.

The significance level was set to *p<*0.003 due to multiple testing.

**Table 4:** Macronutrient composition and intake of selected micronutrients on days with meat dinners and fish dinners (*n*=1,517 participants)

	Days w/red meat dinner^a^ (1,586 days)		Days w/fish dinner^a^ (691 days)		
			
Whole-day intake	Mean^c^	95% CI	Mean^c^	95% CI	*p* b
Energy, MJ/day	9.7	9.6,9.9	9.2	8.9,9.4	<0.001
Protein, E%	17	17,18	19	19,20	<0.001
Fat, E%	36	35,36	34	33,35	<0.001
SFAs, E%	14	14,14	12	12,13	<0.001
MUFAs, E%	12	12,13	11	11,11	<0.001
PUFAs, E%	6	6,6	7	7,7	<0.001
Carbohydrates, E%	43	43,43	42	42,43	0.10
Added sugar, E%	7	7,7	6	6,7	0.002
Fiber, g/MJ	2.6	2.6,2.7	2.8	2.7,2.9	0.001
Alcohol, % of days with intake	22	–	22	–	0.77
Vitamin D, µg/day	4	4,5	11	11,12	<0.001
Folate, µg/day	76	73,78	92	87,96	<0.001
Vitamin C, mg/day	146	136,156	139	126,153	0.39
β-carotene, µg/day	2,165	2,006,2,323	3,948	3,712,4,185	<0.001
Iron, mg/day	12	12,12	10	10,10	<0.001

MJ, mega joule; E%, percentage of energy; SFAs, saturated fatty acids; MUFAs, monounsaturated fatty acids; PUFAs, polyunsaturated fatty acids.

a Red meat dinners were defined as dinners containing at least 30 g of red meat while and less than 30 g of fish, while fish dinners were defined as dinners containing at least 30 g of fish and less than 30 g of meat.

b Linear mixed models for continuous variables and logistic mixed model for alcohol use.

c Means and 95% CIs adjusted for sex, age group, BMI, educational level, interest in a healthy diet, smoking habits, whether the day was normal with regard to food and beverage intake, and weekdays/weekends. For alcohol, the percentage of days with alcohol intake is presented.

The significance level was set to *p<*0.003 due to multiple testing.

## Discussion

To the best of our knowledge, this is the first study to report differences in side dish consumption and nutrient intake between red meat and fish dinners. We found that fish dinners were more likely to include potatoes and carrots, compared with red meat dinners, which were more likely to contain bread, tomato sauce, and cheese. With regard to macronutrient composition, red meat dinners were higher in energy, fat, SFAs, MUFAs, and iron; and lower in protein, PUFAs, folate, β-carotene, and vitamin D than fish dinners. The same pattern was found when comparing the whole-day intakes of the same nutrients on days with red meat versus fish dinners.

Differences in side dish choice between meat and fish dinners have not been an area of focus in the scientific literature. A study from 1999 examined side dish choices for fish and meat dinners for 458 adult Norwegians based on one 24-h recall (14). This previous study and the present study are not directly comparable because of several dissimilarities in the definitions of fish and meat dinners and the methodologies used for collecting dietary data. However, both studies found that potatoes were more common in fish than meat dinners, whereas bread was more common in meat than fish dinners. A recently published study from the Norwegian Women and Cancer Cohort (15) compared high and low consumers of potatoes in a nationally representative sample of women aged 41–70. High consumers of potatoes were defined as those consuming at least two potatoes per day, whereas low consumers consumed one or less than one potato per day. Several differences in dietary intakes were seen between the groups but of special interest in this regard was the higher intake of fish in the high potato consumption group, suggesting a relationship between intake of fish and potatoes similar to what we observed in the present study.

FBDGs from various countries recommend that red meat intake should be reduced in favor of white meat or fish (4). However, it is not known whether changing the main protein source leads to other alterations in dietary intake. Our results show that fish dinners were more often accompanied by vegetables, particularly carrots. Furthermore, energy and SFA intakes were lower for fish than for red meat dinners. These differences were maintained also when looking at whole-day intakes of the same nutrients. Hence, this may suggest that following the recommendation of increased fish intake would lead to more frequent vegetable consumption, and lower total energy and SFAs intakes. From a nutritional point of view, these changes would be regarded as beneficial, possibly contributing to the gain in nutritional quality of switching from red meat to fish. This is of course a simplified interpretation because we do not know whether those who begin eating more fish dinners will choose the same amounts of fish or the same side dishes as those who already are fish eaters.

The choice of side dishes may not always be up to the individual himself or herself when eating outside of home. However, only 6% of the dinners included in the present analyses were eaten at restaurants, cafeterias, or fast food outlets; whereas 81% of the dinners were consumed at home. Hence, the influence of restaurant dinners and dinners consumed in other out-of-home locations was rather small in our sample.

Associations between the intakes of different protein sources and various side dishes may vary among different cultures. However, a positive association between fish and vegetable intakes also has been reported in other populations (16–20), suggesting that this association may have international relevance. Information about foods that are eaten together is also important when studying associations between dietary factors and health (16, 20). A diet high in one food, for instance red meat or fish, may also contain high levels of other associated foods that may or may not contribute to the effect in question. Neglecting to take the associated foods into account may therefore lead to an over- or underestimation of the studied association.

As expected, vitamin D intake was considerably higher for fish versus meat dinners, and this difference was also observed for whole-day vitamin D intake on days with fish dinners and on days with red meat dinners. Vitamin D intake and status have previously been reported to be low in Norwegian adults (21); hence, inclusion of more fish dinners, particularly dinners with oily fish in the diet, would be beneficial also for this reason. It should be noted that vitamin D intake from red meat dinners was most likely somewhat underestimated in this study because the version of the Norwegian Food Composition Table used for nutrient calculations did not include vitamin D values for meat and meat products. Several food composition tables have included vitamin D values for meats, typically ranging from 0.1–1.0 µg/100 g meat (22, 23). However, inclusion of 0.1–1 µg vitamin D/100 g meat would only add a small amount to the vitamin D intake for meat dinners compared to fish dinners. Red meat is a source of iron, and iron intake was significantly higher for the red meat dinners than from fish dinners. Still, the difference in whole-day intake of iron on days with red meat versus fish dinners was not as pronounced as what was observed for vitamin D, most likely because there are more dietary sources of iron than of vitamin D. Quite a noticeable difference in β-carotene intake was seen between red meat and fish dinners; this difference was largely caused by a higher intake of carrots from fish dinners than from red meat dinners.

Even though fish intake in Norway traditionally has been and still is higher than in many other countries (5), both our data and previous dietary surveys (6, 7) show that fish intake is much lower than the intake of meat. As seen from the results presented herein, the number of red meat dinners was more than twice as high as the number of fish dinners. One of the potential barriers to increasing fish consumption may be concerns about unwanted substances in fish (24), such as methylmercury, dioxins, and dioxin-like PCBs. In 2011 and 2014, the Norwegian National Institute for Consumer Research conducted web-based surveys that included questions about which factors the respondents regarded as limiting to increasing their intake of fish (25). The alternatives included high price, poor selection, bad quality, poor knowledge about preparation of fish, preference for meat, and skepticism toward the production methods. The respondents were free to choose more than one factor. In both surveys the top two reported factors limiting an increased fish intake were that the respondents preferred meat and that the selection of fish was too poor. Price was the third limiting factor. Skepticism toward the production methods was regarded as a limiting factor by 25% of the respondents in 2011 and by 29% in 2014. Levels of contaminants in fish were not specifically mentioned in these web surveys, but concern about such compounds may have been one of the reasons for the reported concern about the production methods. However, the most recent risk assessment performed by the Norwegian Scientific Committee for Food Safety (26) concluded that in the Norwegian society, the benefits of the recommended fish consumption clearly outweigh the negligible risk presented by current levels of contaminants and other known undesirable substances in fish. With regard to side dish choice, our results from the present comparison do not suggest any negative dietary effects from switching from red meat to fish as the main protein source of the dinner meal. However, more information both concerning reasons for choosing and not choosing fish and what real-life replacements are made when persons switch from meat to fish is needed.

### Strengths and limitations

Important strengths of the Norkost 3 study include the detailed descriptions of foods, portion sizes, and meal types. In addition to providing information about which foods that have been consumed, the 24-h recall methodology offers the possibility of studying how foods are combined in individual meals.

Although the sample size of the study was fairly large, the low participation rate of 37% limits the generalizability of our results. Those who chose not to participate may consume dinners with a different composition than what was reported by the participants. A higher proportion of the participants in the Norkost 3 study had a higher education compared to the general population (12). Because more highly educated individuals often have been found to have healthier dietary habits (27), a somewhat higher frequency of consumption of healthier side dishes may have been observed than what would have been the case in the general population. However, this is likely to affect both fish and red meat dinners as the percentages of participants with higher education were similar among the consumers of fish dinners and the consumers of red meat dinners. To eliminate a potential effect of differences in personal characteristics between those consuming fish dinners and those consuming red meat dinners, a similar comparison to the one presented herein was made for those participants who had consumed one red meat dinner and one fish dinner during the 2 recall days. This comparison included 540 dinners from 270 participants. The findings were generally the same regarding both the use of side dishes and differences in nutrient content between the two dinner types. However, no differences were seen in intakes of added sugars and fiber between red meat and fish dinners in the last comparison.

The calculation of BMI in the present study was based on self-reported weight and height. As self-reported weight might be prone to underreporting (28, 29), this might have led to an underestimation of the proportion of participants with a BMI >25 kg/m^2^. However, because BMI was treated as a covariate rather than a dependent variable, this limitation is not likely to be a major source of error.

## Conclusions

Fish dinners were accompanied by different side dishes than red meat dinners. With regard to nutrient content, fish dinners generally had a healthier profile than red meat dinners. However, iron intake was higher for red meat dinners. Information about associated foods will be useful both for developing public health guidelines and when studying associations between dietary factors and health outcomes.
